# Quantitative proteome and phosphoproteome datasets of DNA replication and mitosis in *Saccharomyces cerevisiae*

**DOI:** 10.1016/j.dib.2022.108741

**Published:** 2022-11-09

**Authors:** Valentina Rossio, Joao A Paulo

**Affiliations:** Department of Cell Biology, Blavatnik Institute at Harvard Medical School, Boston, MA 02115, USA

**Keywords:** Cell cycle, Phosphorylation, DNA replication, Mitosis, Yeast, TMT, Isobaric tagging

## Abstract

Cell division is a highly regulated process that secures the generation of healthy progeny in all organisms, from yeast to human. Dysregulation of this process can lead to uncontrolled cell proliferation and genomic instability, both which are hallmarks of cancer. Cell cycle progression is dictated by a complex network of kinases and phosphatases. These enzymes act on their substrates in a highly specific temporal manner ensuring that the process of cell division is unidirectional and irreversible. Key events of the cell cycle, such as duplication of genetic material and its redistribution to daughter cells, occur in S-phase and mitosis, respectively. Deciphering the dynamics of phosphorylation/dephosphorylation events during these cell cycle phases is important. Here, we showcase a quantitative proteomic and phosphoproteomic mass spectrometry dataset that profiles both early and late phosphorylation events and associated proteome alterations that occur during S-phase and mitotic arrest in the model organism *S. cerevisiae*. This dataset is of broad interest as the molecular mechanisms governing cell cycle progression are conserved throughout evolution. The data has been deposited in ProteomeXchange with the dataset identifier PXD037291.

**Specifications Table**

**Table:** 

Subject:	Omics: Proteomics
Specific subject area:	Yeast Phosphoproteomics, cell cycle
Type of data:	Figure Excel Tables RAW mass spectrometry data files in ThermoFisher format
How the data were acquired:	Database searches were performed using Comet (2022.01 rev. 0). Data were acquired with Xcalibur 4.1 and Tune 3.5.The RAW files were converted to mzXML using MSconvert 3.0. Mass spectrometric data were acquired on an Orbitrap Fusion Lumos or Eclipse mass spectrometer with a Proxeon NanoLC-1200 UHPLC (ThermoFisher Scientific).
Data format:	Raw Analyzed
Description of data collection:	*S. cerevisiae* cells were released from G1 arrest in fresh medium with or without nocodazole. Samples were collected after 30 and 40 min from the release in fresh medium and 90 min after release in nocodazole. The DNA content of the cells was analyzed by FACS analysis. Digested samples were enriched for phosphopeptides and labeled with tandem mass tag (TMTpro) reagents. The pooled sample was fractionated prior to MS analysis.
**Data source location**	Harvard Medical School Boston, MA USA
**Data accessibility**	Mass spectrometry data have been deposited in ProteomeXchange via the PRIDE repository. Pride Data identification number: PXD037291 Direct URL to data: http://proteomecentral.proteomexchange.org/cgi/GetDataset?ID=PXD037291 Instructions for accessing these data: Data has been made public

## Value of the Data

• These datasets are a valuable resource for understanding the mechanisms regulating the cell cycle that are deregulated in cancer cells
• These datasets provide hundreds of targets of kinases/phosphatases that are useful for those studying the cell cycle, cancer biology or how these enzymes function.
• Because anticancer drugs targeting cell cycle kinases and phosphatases are currently used in the clinic, these data can help develop novel chemotherapeutic drugs.

## 1 Data Description

**Fig. 1:**
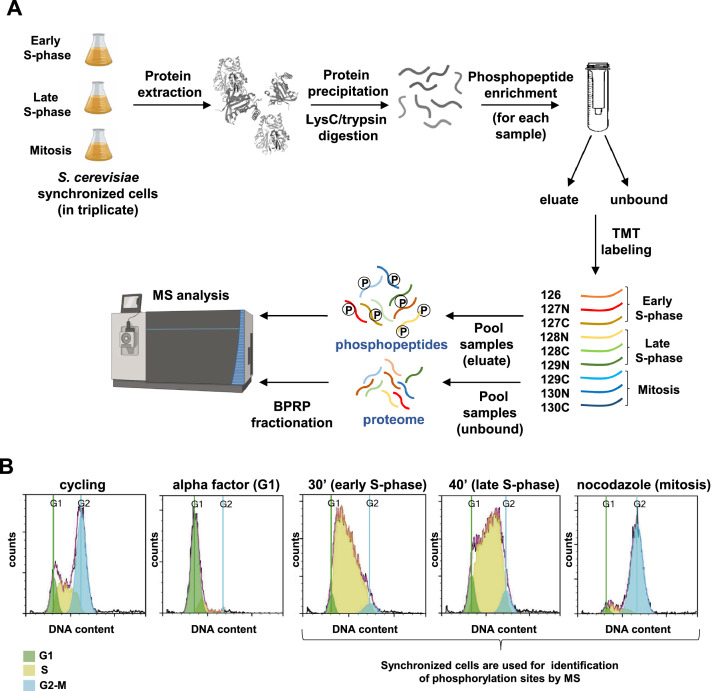
Experimental workflow and cell cycle synchronization A) Synchronized yeast cells were collected and lysed. Proteins were extracted, precipitated and digested with LysC followed by trypsin. The peptides obtained from the digestion were subjected to phosphopeptide enrichment. Both the eluate (phosphopeptides) and the flow-through from this enrichment were separately labeled with unique tandem mass tag (TMT) reagents and mixed 1:1. The mixed flow-through sample was fractionated by basic pH reversed-phase (BPRP) HPLC. The collected peptides fractions and the phosphopeptides were analyzed by mass spectrometry (MS). This figure has been created in part using Biorender.com **B)** FACS analysis of the DNA content of the yeast cells collected for the mass spectrometry analysis are shown.

This article includes both the RAW data files (*.raw), the database search engine results (*.mzIdentML), and the processed data from a mass spectrometry-based multiplexed proteomics and phosphoproteomics experiment investigating yeast cells synchronized at different stages of the cell cycle. These data are shared in the PRIDE repository that is part of the ProteomeXchange Consortium and are accessible using the identifier PXD037291. Fig. 1 depicts cell cycle synchronization, preparation of the samples for LC-MS/MS analysis (Fig. 1A) and FACS analysis of DNA content (Fig. 1B).

Supplemental Table 1 lists the RAW files, the associated experiment, the instrument used for data acquisition, the compensation voltage (CV) sets used, and the chromatographic gradient length. Supplemental Table 2 lists the protein identifications. Columns include: Protein ID, gene symbol, description, protein group ID, number of peptides assigned to a given protein, 9 columns of TMT signal-to-noise values, 9 columns of TMT signal-to-noise values that have been scaled to 100 across all channels. Supplemental Table 3 lists the phosphopeptide identifications. Columns include: Peptide sequence, 9 columns of TMT signal-to-noise values, assigned protein ID, phosphorylation site residue, phosphorylation site position, phosphorylation site motif (phosphorylation site plus six flanking residues), the A-score (>13 is significantly localized), redundancy of the peptide in the proteome, and the file of origin. Supplemental Table 4 lists the phosphorylation sites. Columns include: type of site (single or multiple sites per peptide), assigned protein ID, gene symbol, protein description, phosphorylation site residue, phosphorylation site position, phosphorylation site motif (phosphorylation site plus six flanking residues), the A-score, redundancy of the peptide in the proteome (U=unique, R=redundant), the peptide sequence, 9 columns of TMT signal-to-noise values for the phosphorylation site, 9 columns of TMT signal-to-noise values that have been scaled to 100 across all channels for the phosphorylation site, 9 columns of TMT signal-to-noise values that have been scaled to 100 across all channels for the corresponding protein, and the correlation value for the phosphorylation site and corresponding protein abundance (which ranges from -1 to 1).

## 2 Experimental Design, Materials and Methods

### 2.1 Yeast growth and cell cycle synchronization

Wild type *S. cerevisiae* cells (W303 background: *ade2‐1, trp1‐1, leu2‐3,112, his3‐11, 15, ura3*) were grown at 25°C in YEP medium (1% yeast extract, 2% bactopeptone, 50 mg/l adenine) supplemented with 2% glucose as the carbon source (YEPD). Triplicate cultures were synchronized in G1 with alpha factor (4 mg/ml) at 25°C for 2.25 hr. After the arrest, yeast cells were washed with YEPD by centrifugation at 2,000 *g* and subsequently released in fresh YEPD (for cell cycle progression through S-phase) or in YEPD containing the microtubule depolymerizing drug nocodazole (mitotic arrest) at 25°C. Nocodazole was used at 15 μg/ml. Cells were harvested after 30 min (early S-phase) and 40 min (late S-phase) in the absence of nocodazole or after 90 min in nocodazole-containing medium. Cells were collected by centrifugation (2,000 *g)* and rinsed with 1 ml of 20% TCA for downstream mass spectrometry-based proteome and phosphoproteome profiling. In parallel, an aliquot (2 ml) of cells was collected for FACS analysis of DNA content (see 2.2).

### 2.2 FACS analysis of DNA content

Flow cytometric DNA quantitation was performed as reported previously [1]. In brief, cells were collected at 10,000 *g* and fixed with 1 ml of 70% ethanol for at least half an hour. After fixation, cells were washed with 50 mM Tris–HCl pH 7.5, resuspended in 0.5 ml of 50 mM Tris–HCl pH 7.5 containing 0.5 mg/ml RNAse and incubated at 37°C overnight. The next morning, cells were centrifuged and resuspended in a freshly prepared solution of 5 mg/ml pepsin dissolved in 55 mM HCl. Samples were incubated at 37°C for 30 min. After pepsin treatment, cells were washed with FACS buffer (200 mM Tris–Cl pH 7.5, 200 mM NaCl, 78 mM MgCl_2_) and resuspended in FACS buffer supplemented with 50 μg/ml propidium iodide. After sonicating briefly (10-15 seconds), the samples were diluted in 50 mM Tris–HCl pH 7.5 and analyzed with a NovoCyte flow cytometer (ACEA).

### 2.3 Sample processing for quantitative TMT-proteomic analysis

Samples were prepared following the SL-TMT protocol [2]. Yeast pellets were resuspended in lysis buffer (8 M urea in 200 mM EPPS, pH 8.5 supplemented with protease and phosphatase inhibitors) and lysed mechanically. A standard BCA assay was performed to determine the protein concentration of each sample. Reduction of lysates (5 mM TCEP for 15 min.) was followed by alkylation (10 mM iodoacetamide for 30 min.) and quenching (5 mM DTT for 15 min.).

A total of 2 mg of protein from each sample was chloroform-methanol precipitated. The precipitated proteins were resuspended in 200 mM EPPS pH 8.5, digested first by Lys-C overnight at room temperature and later by trypsin (6 h at 37°C). Both enzymes were used at a 1:100 enzyme-to-protein ratio.

To enrich phosphopeptides, each sample was desalted over a 200 mg SepPak column and phosphopeptides were enriched with the Pierce High-Select Fe-NTA Phosphopeptide enrichment kit following manufacturer's instructions. The washes and the unbound fraction of this enrichment were desalted, and 50 mg of each sample was used for whole proteome level analysis.

The samples were then labeled with tandem mass tag (TMTpro) reagents [3] as follows: early S-phase triplicates: 126,127n, 127c; late S-phase triplicates:128n, 128c, 129n; mitosis triplicates:129c, 130n, 130c. Acetonitrile was added to a final volume of 30% prior to adding the TMTpro labeling reagent. For protein level analysis, ∼50 µg of peptides were labeled with 100 µg of TMT. For phosphopeptide analysis, we estimated the phosphopeptide enrichment to be ∼1.5:100 and so ∼30 µg of peptides were labeled with 60 µg of TMT. Labeling occurred at room temperature for 1h. ∼2 µg of peptide from each sample was pooled, desalted and analyzed by mass spectrometry to check labeling efficiency.

TMT labeling efficiency was determined using the “label-check” sample, which is a quality control step prior to the final pooling of the TMT-labeled samples. Here, we combined a small amount (1-3 µL or ∼2µg) of each sample and analyzed it by mass spectrometry to confirm that the protein digestion was successful, if the degree of labeling was sufficient, and if the labeled samples contained approximately equal amount of peptides. During database searching, the TMTpro label was considered a variable modification at the N-terminus and at lysine residues. We then determined the labeling efficiency for the N-terminus and the lysine residues by dividing labeled N-terminal peptides by total peptides, and also the labeled lysine-terminating peptides by the total lysine-terminating peptides. The labeling efficiency should be greater than 95% before proceeding with the analysis. Once labeling efficiency was verified (here, >97%), hydroxylamine was added at a final concentration of ∼0.3% and incubated for 15 min. at room temperature. The remaining samples were pooled at a 1:1 ratio across all channels.

For proteome-level analysis, the pooled samples were desalted by solid phase extraction (100 mg Sep-Pak column) and fractionated using basic-pH reversed-phase (BPRP) Liquid Chromatography. An Agilent 1200 pump with an Agilent 300Extend C18 column (2.1 mm ID, 3.5 μm particles, and 250 mm in length) was used for fractionation. The flow rate over the column was 0.25 mL/min and we used 50-min linear gradient with 5% to 35% acetonitrile in 10 mM ammonium bicarbonate pH 8. Ninety-six fractions were collected and concatenated into 24 superfractions prior to desalting [3]. These 24 superfractions were sub-divided into two groups, each consisting of 12 non-adjacent superfractions. These superfractions were subsequently acidified with 1% formic acid and vacuum centrifuged to near dryness. Each superfraction was desalted via StageTip [4]. Once dried by vacuum centrifugation, the sample was reconstituted using 5% formic acid and 5% acetonitrile prior to acquisition of LC-MS/MS data.

For phosphoproteome-level analysis, the pooled phosphopeptide sample was fractionated using the Pierce High pH Reversed-Phase Peptide Fractionation Kit. Twelve fractions were collected using: 5%, 10%, 12.5%, 15%, 17.5%, 20%, 22.5%, 25%, 30%, 35%, 40%, and 80% acetonitrile in 0.1% triethylamine buffer. The fractions were concatenated into 6 by mixing the eluates as follows: 5% and 22.5%, 10% and 25%, 12.5% and 30%, 15% and 35%, 17.5% and 40%, and 20% and 80%. These superfractions were subsequently acidified with 1% formic acid and vacuum centrifuged to near dryness. Each superfraction was desalted via StageTip, dried again by vacuum centrifugation, and reconstituted in 5% acetonitrile, 5% formic acid for LC-MS/MS data acquisition. The phosphorylation efficiency was determined by dividing the number of phosphorylated peptides by the total number of peptides identified in a given phosphopeptide enrichment. Phosphorylation efficiency across each fraction ranged between 90-95%, which is similar to the values achieved previously [5] .

### 2.4 Mass spectrometry data collection and processing

Mass spectrometric data were collected on an Orbitrap Fusion Lumos or Orbitrap Eclipse mass spectrometer, each which were coupled to a Proxeon NanoLC-1200 UHPLC and a FAIMSpro interface [6]. A 100 μm capillary column was packed with 35 cm of Accucore 150 resin (2.6 μm, 150 Å; ThermoFisher Scientific) and used for on-line peptide fractionation.

A total of 36 RAW files were collected for the whole proteome level dataset to increase the diversity of peptide population. Data for the first subset of 12 superfractions were acquired on an Orbitrap Lumos with a CV set of -40/-60/-80V, while the subset of the other 12 superfractions were acquired with a CV set of -50/-75V over a 90 min gradient. The remnants of each superfraction were combined so that each new superfraction (n=12) consisted of 8 fractions that were collected down the column of the 96-well elution plate. This new set of 12 samples were analyzed on an Orbitrap Eclipse over a 120 min gradient with a CV set of -40/-60/-80V. A one second TopSpeed cycle was used for each CV. The scan sequence began with an MS1 spectrum (Orbitrap analysis, resolution 60,000, 400–1600 Th, automatic gain control (AGC) target 100%, maximum injection time was set to auto) followed by the hrMS2 stage that consists of fragmentation by higher energy collisional dissociation (HCD, normalized collision energy 37%) and analysis using the Orbitrap (AGC 250%, maximum injection time 86 ms, isolation window 0.5 Th, resolution 50,000) on an Orbitrap Fusion Lumos mass spectrometer. Similarly, on the Orbitrap Eclipse, the hrMS2 stage consisted of fragmentation by higher energy collisional dissociation (HCD, normalized collision energy 36%) and analysis using the Orbitrap (AGC 200%, maximum injection time 86 ms, isolation window 0.7 Th, resolution 50,000).

A total of 17 samples were analyzed for the phosphoproteomic dataset. Data for each of the 6 superfractions were collected on an Orbitrap Fusion Lumos with a CV set of -40/-60/-80V over a 90 min gradient. Five of these samples were reanalyzed with a CV set of -50/-65V, while the remaining sample was analyzed with a CV set of -35/-50/-65V also over a 90 min gradient. Also, the combined flowthrough and wash fraction was analyzed twice over a 90 min gradient (once with a CV set -40/-60/-80V and the second time with a CV set -35/-50/-65V). In addition, as in the proteome level dataset, the remnants of the 6 superfractions were combined, and three 150 min analyses were performed, two with CV sets -35/-50/-65V and another with set -50/-65V. For all samples, the scan sequence began with an MS1 spectrum (Orbitrap analysis, resolution 120,000, 400–1500 Th, automatic gain control (AGC) target 100%, maximum injection time was set to auto). The hrMS2 stage consisted of fragmentation by higher energy collisional dissociation (HCD, normalized collision energy 36%) and analysis using the Orbitrap (AGC 300%, maximum injection time 250 ms, isolation window 0.5 Th, resolution 50,000).

Once the spectra were converted to mzXML using MSconvert [7], database searching could be performed. For this database, we included all entries from the Saccharomyces Genome Database (SGD; August 2021) which was concatenated with a version of the database in which the order of the amino acid residues of each protein was reversed. Our forward database has 6077 reviewed yeast entries and 115 common contaminants. Database searches used a 50-ppm precursor ion tolerance and a product ion tolerance of 0.03 Da. We have traditionally used the 50ppm mass tolerance for our Sequest, and now Comet, database searches. These wide mass tolerance windows were chosen to maximize sensitivity in conjunction with Comet searches and linear discriminant analysis [8]. For static modifications, lysine residues and peptide N-termini were modified with +304.207 Da representing the TMTpro labels. Meanwhile, all cysteine residues were modified with iodoacetamide (carbamidomethylation) that results in a +57.021 Da increase in mass. Also, methionine oxidation (+15.995 Da) was set as a variable modification. Likewise, deamidation (+0.984 Da) at glutamine and asparagine residues and phosphorylation (+79.966 Da) at serine, threonine, and tyrosine residues were also set as variable modifications for phosphopeptide enrichment. The false discovery rate (FDR) was set at 1% at the peptide level with filtering using linear discriminant analysis [9,10]. The protein lists were assembled further to a final protein-level FDR of 1% [11]. The intensities of reporter ions were corrected for the isotopic impurities of the different TMT reagents [11]. For each protein, the peptide signal-to-noise (S/N) measurements were summed and normalized to account for equal protein loading by equating the sum of the signal for all proteins in each channel.

## Ethics Statement

This study does not include work with human subjects, animal experiments or data collected from social media platforms

## CRediT Author Statement

**Valentina Rossio:** Conceptualization, Writing – original draft preparation, Investigation; **Joao A. Paulo:** Conceptualization, Data curation, Writing – original draft preparation, Resources, Software.

## Declaration of Competing Interest

The authors declare that they have no known competing financial interests or personal relationships that could have appeared to influence the work reported in this paper.

## Data Availability

Quantitative proteome and phosphoproteome datasets of DNA replication and mitosis in Saccharomyces cerevisiae (Original data) (Pride).

## Appendix Supplementary materials

Image, application 1

Image, application 2

Image, application 3

Image, application 4
